# Recent Advances in Pd-Decorated SnO_2_ Nanowires Toward Room-Temperature Methane Sensing: A Mini-Review of Synthesis Strategies, Catalytic Mechanisms, and Mining Safety Applications

**DOI:** 10.3390/nano16161017

**Published:** 2026-08-18

**Authors:** Moses Mpofana Radebe, Xoliswa Cingo, Hillie Kenneth Thembela

**Affiliations:** Department of Physics, School of Science, Engineering and Technology, University of South Africa, Pretoria 0002, South Africa

**Keywords:** Pd-decorated SnO_2_, nanowires, room-temperature methane sensor, spillover mechanism, Schottky barrier, mining safety, IoT gas detection, chemiresistive sensor, nanowire gas sensor, selectivity, bimetallic decoration, interfering gas discrimination

## Abstract

Strict monitoring of methane (CH_4_) during underground coal mining is necessary, as the lower explosive limit (LEL) is 5 vol% in air. A conventional tin oxide (SnO_2_)-based metal–oxide semiconductor (MOS) sensor has an operating temperature of 200–400 °C, which requires a prohibitive power demand and entails the risk of ignition within an intrinsically safe environment. The decoration of SnO_2_ nanoarchitectures with palladium has been demonstrated to achieve room temperature (RT) detection of CH_4_ due to the chemical sensitisation spillover mechanism and electronic sensitisation by Schottky barrier modulation. Moreover, palladisation of SnO_2_ nanowires (NWs) is likely to be an effective route for achieving a more efficient detection of CH_4_ aerosol at RT or near RT. The purpose of this mini-review is to provide a critical synthesis of advances that have been reported between 2020 and 2026. Because no published study to date has directly demonstrated room-temperature CH_4_ detection using pure Pd-decorated SnO_2_ nanowires, performance data from mechanistically analogous systems—namely H_2_-sensing Pd–SnO_2_ nanowires and CH_4_-sensing non-nanowire Pd–SnO_2_ nanostructures—are included in this review and are explicitly labelled as such throughout. This absence of direct RT CH_4_ NW data constitutes the primary research gap motivating this review. The performance of Pd-containing SnO_2_ nanostructures reported in the literature spans response values of 17.6 (300 ppm CH_4_, 2.5 mol% Pd–SnO_2_ nanoporous, 340 °C) to 21.3 (3000 ppm CH_4_, bimetallic Pt–Pd–SnO_2_ mesoporous, 400 °C), representing a 3–10× improvement over bare SnO_2_ (response: 2–10 in the same concentration range). These benchmarks were obtained at elevated temperatures (340–400 °C); no equivalent room-temperature CH_4_ detection data for Pd–SnO_2_ nanowires currently exists in the published literature. Reported response times range from 3 to 9 s at elevated temperature (340–400 °C) to 74–78 s for room-temperature visible-light-activated systems, where photocatalytic oxygen activation is the rate-limiting step. The 30 s MSHA alarm threshold is met by elevated-temperature systems but remains a challenge for RT configurations. The LODs were 175.9 ppb (bimetallic PdxPt/SnO_2_ mesoporous system). Two hybrid composites containing rGO exhibited an extended capability for RT operation. Bimetallic PdPt decoration and ML-augmented sensor arrays are identified as the most promising near-term pathways to bridge the selectivity and stability gaps for certified mining deployment.

## 1. Introduction

**Figure 1:**
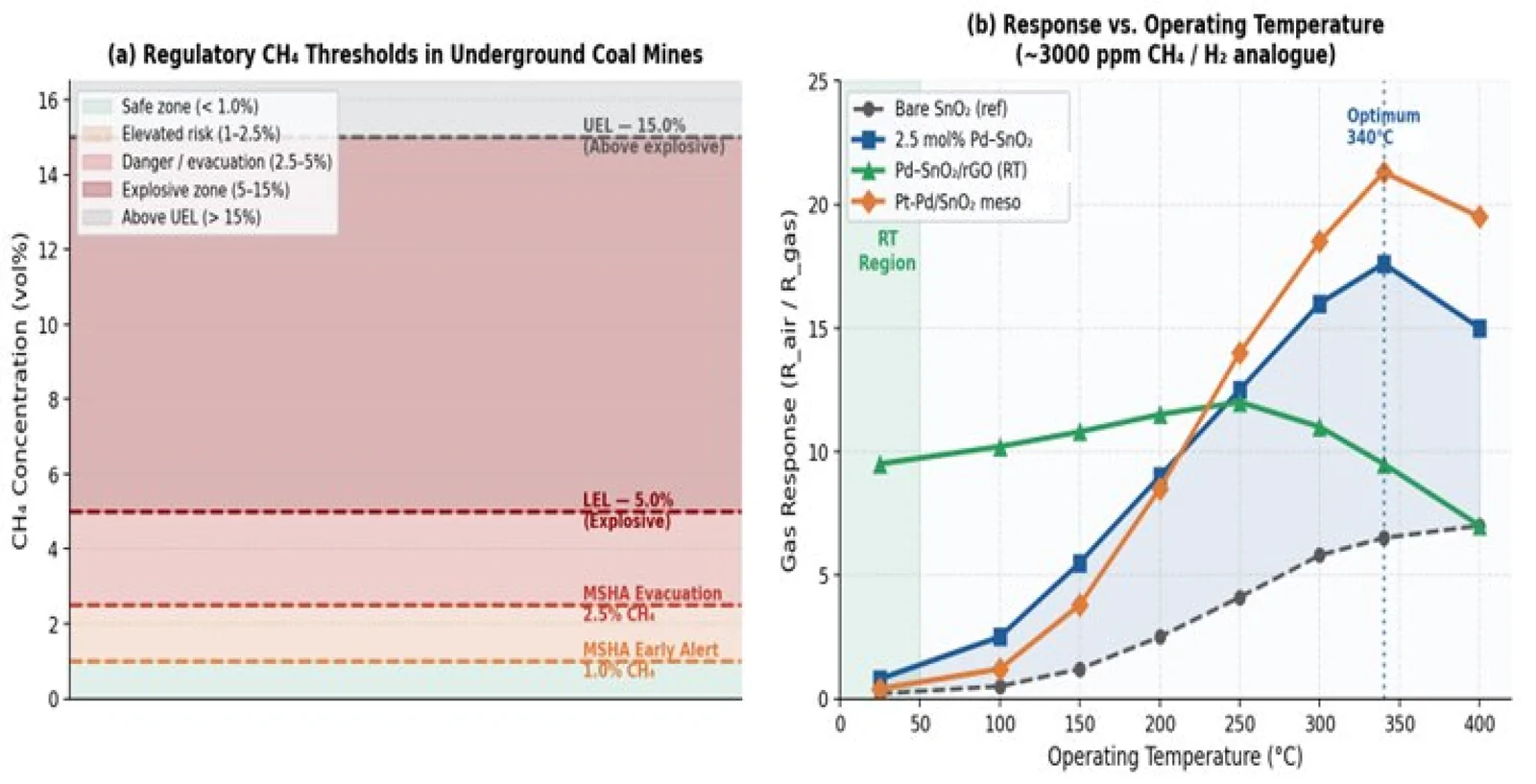
(**a**) Regulatory CH_4_ hazard zones with MSHA and LEL thresholds. (**b**) Response vs. operating temperature for bare SnO_2_, Pd–SnO_2_ nanoporous [1], Pd–SnO_2_/rGO [2], and Pt–Pd/SnO_2_ [3] at 300–3000 ppm CH_4_/H_2_.

Methane is the most prevalent and hazardous gas in underground coal mines. It is classified as a Group 1 explosive gas and forms flammable mixtures in air at concentrations of 5–15 vol%. Methane explosions have caused hundreds of fatalities in mining incidents globally and continue to represent the single largest safety risk in underground coal operations. The United States Mine Safety and Health Administration (MSHA) requires de-energisation of electrical equipment and enhanced ventilation when CH_4_ reaches 1.0%, withdrawal of persons from working places at 1.5%, and immediate extraction of all personnel at 2.0% (30 CFR §75.323)—all thresholds well below the 5% LEL. The sensor system must therefore monitor CH_4_ continuously, rapidly, and reliably while withstanding the harsh conditions inside underground coal mines. Underground conditions are characterised by high humidity, about 50–90% RH, dusty loading, vibration, and very limited access to mains power. Figure 1a illustrates the regulatory CH_4_ hazard zones relative to the LEL, and Figure 1b compares the response–temperature profiles of bare SnO_2_, Pd–SnO_2_ nanoporous [1], Pd–SnO_2_/rGO [2], and Pt-Pd/SnO_2_ [3] systems across the 300–3000 ppm CH_4_/H_2_ range.

For decades, tin oxide (SnO_2_)-based metal–oxide semiconductor (MOS) sensors have dominated the commercial sensing landscape due to their high sensitivity, low manufacturing cost, good mechanical strength, and compatibility with standard microfabrication processes. SnO_2_-based MOS sensors have been extensively investigated for a wide range of target analytes; notably, Ji et al. [4] demonstrated that dynamic measurement combined with principal component analysis (PCA) enables qualitative and quantitative discrimination of SnO_2_ sensor responses across overlapping gas mixtures—a multivariate selectivity methodology directly applicable to CH_4_ monitoring in complex mine atmospheres containing CO, H_2_, and CO_2_. SnO_2_ sensors have since been demonstrated for H_2_ [5] and CH_4_ [1,3] detection, cementing their position as the dominant chemiresistive sensing platform. The basic sensing principle of *n*-type SnO_2_ is oxygen adsorption on the surface. O_2_ from the atmosphere adsorbs on the semiconductor surface and captures conduction-band electrons, resulting in the formation of ionosorbed species (O_2_^−^, O^−^, and O^2−^). Consequently, a depletion layer forms at the surface of the semiconductor, which increases the sensor resistance. When a reducing gas such as CH_4_ is introduced, the surface species react with and consume the adsorbed oxygen.

Pure SnO_2_ sensors are commercially available, but they require high working temperatures (200–400 °C) to furnish sufficient thermal energy to cleave the strong C–H bond in methane (with bond dissociation energy ≈ 439 kJ mol^−1^). These elevated operating temperatures translate to heater power consumption of 0.5–2 W, shortened device lifetimes due to thermal cycling fatigue, and—most critically in mining environments—an exposed hot filament that may itself serve as an ignition source. Research is being conducted to create a catalytic strategy that will lessen the activation barrier for CH_4_ oxidation, subsequently allowing sub-200 °C or even room temperature sensing.

The application of palladium (Pd) has become popular as a noble metal dopant for SnO_2_ gas sensors. In the case of a single-metal configuration, palladium (Pd) has one of the highest catalytic activities for low-temperature CH_4_ oxidation among the platinum-group metals. It activates C–H bond breaking via surface-mediated dissociative chemisorption even at temperatures below 100 °C when in the form of nanoparticles on high-surface-area supports. When Pd is deposited on SnO_2_, it acts via two complementary pathways. These pathways are mutually reinforcing. First, there is chemical sensitisation. Catalytically dissociated hydrogen and hydrocarbon radical species spill over onto adjacent SnO_2_ surface sites. Chemical sensitisation greatly accelerates oxidation kinetics. Second, there is electronic sensitisation. The work function mismatch between Pd (φ ≈ 5.1–5.6 eV) and SnO_2_ (φ ≈ 4.5 eV) establishes a Schottky barrier at the metal–semiconductor interface. This also modulates the charge carrier density. Lastly, it provides enhanced resistance to gas exposure.

The sensing properties of bulk materials or thin films are not as good as those of one-dimensional (1D) nanowire architectures of SnO_2_. These nanowires have several intrinsic advantages. A high density of active sites for surface oxygen adsorption and Pd-catalysed CH4 activation is created because of the ultra-high surface-to-volume ratios. Another is how the geometry of the nanowire encourages directional charge transport in the 1D channel. This completely removes the grain-boundary conduction losses associated with polycrystalline films. It also allows for the easy and fast diffusion of the analyte to the sensing surface. Another concern of interest is how SnO_2_ NW diameters (typically 50–200 nm) compare with twice the depletion layer width (~10–30 nm for SnO_2_ under typical sensing conditions). This means that when the layer-width criterion is satisfied, the variations in the gas-response (resistance) are fully transduced over the full wire cross-section and are maximal.

Noble-metal-decorated SnO_2_ sensors have been reviewed in several high-impact publications recently. Zhu et al. [6] give a wide-ranging overview of noble-metal-decorated metal oxide nanomaterials for chemiresistive sensing; Jiao et al. [7] survey conductive-type methane sensors more generally. This review is distinguished from prior works in three ways: (i) it focuses entirely on Pd as the sensitiser, explicitly benchmarking Pd chemistry against Pt and Au analogues; (ii) it emphasises NW morphology and quantifies the performance difference between NW-specific and non-NW data—a distinction absent from prior reviews; and (iii) it incorporates explicit assessment against MSHA, ATEX, and AQ regulatory frameworks, an approach not present in prior sensor-materials reviews. According to a recent review on gas nanosensors for mining applications by Baharfar et al. [8], the Pd–SnO_2_ NW systems are not dealt with in isolation, the RT CH_4_ NW performance gap is not quantified and the spillover and Schottky barrier modulation are mechanistically very shallow. This review clearly indicates that observations are based on non-NW/CH_4_ data instead of suggesting that it is equivalent.

This mini-review surveys the literature published from 2020 to 2026 and is organised into the following sections: (i) recent advances in synthesis strategies; (ii) catalytic and electronic sensing mechanisms with mechanistic evidence; (iii) quantitative performance benchmarking; (iv) integration pathways to ensure safe mining operations; and finally (v) challenges and future directions.

## 2. Synthesis Strategies for Pd-Decorated SnO_2_ Nanowires

**Figure 2:**
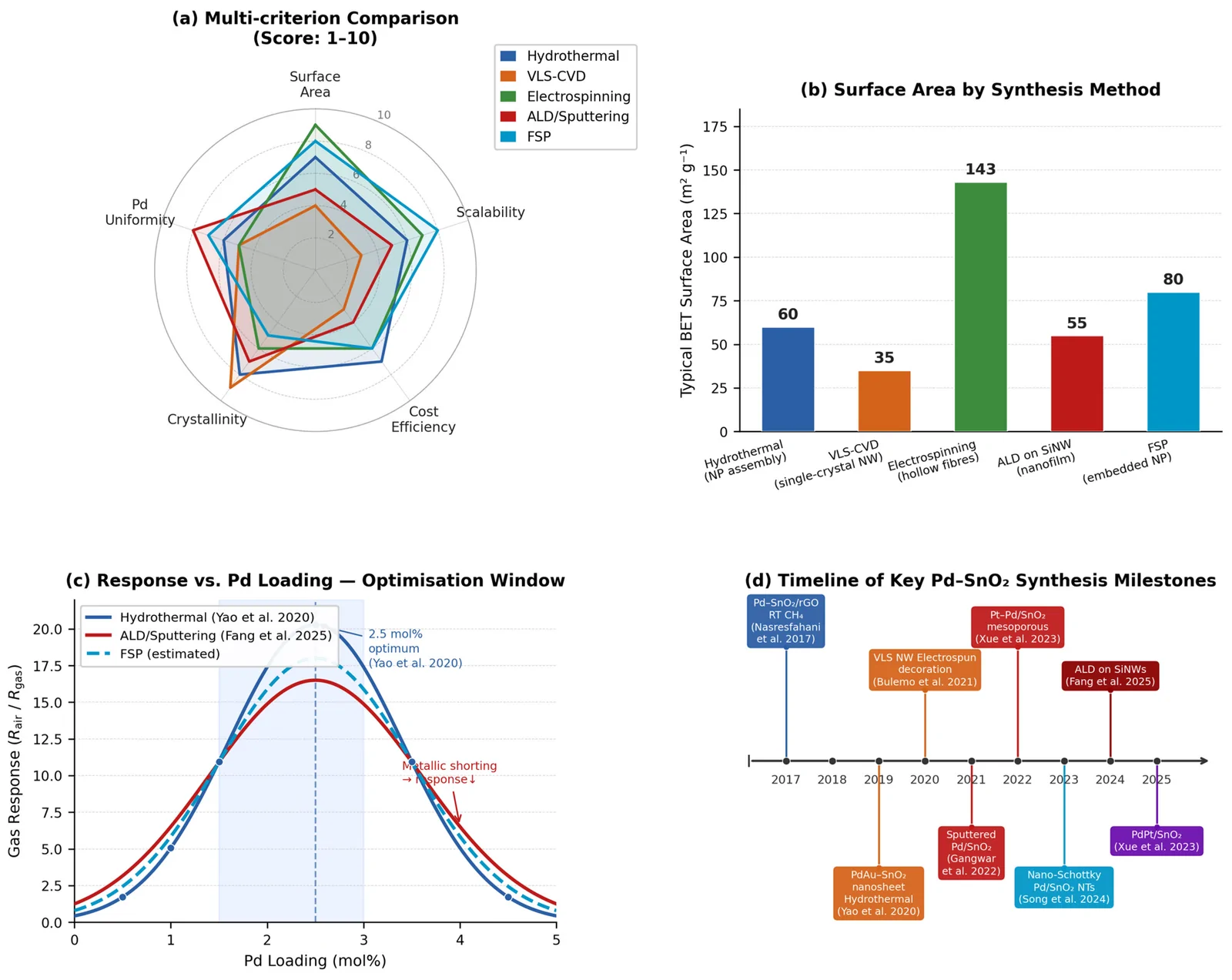
(**a**) Radar multi-criterion synthesis scoring. (**b**) BET surface area by method. (**c**) Response vs. Pd loading with optimal window and metallic-shorting regime. (**d**) Milestone timeline, 2017–2025.

The catalytic and sensing characteristics of Pd–SnO_2_ NW composites are a strong function of a triplet. The morphology of SnO_2_ nanowires, including their diameter, crystal structure, porosity, and surface area. Also, the size of Pd nanoparticles, the density of their distribution and their oxidation state. Ultimately, the quality of the contact between Pd and SnO_2_. Simultaneously controlling all three parameters across synthesis scales from milligram (laboratory) to kilogram (industrial) remains the central challenge in this field. These routes broadly fall into two categories—SnO_2_ NW formation and subsequent Pd post-decoration—though one-pot approaches combining both steps have gained traction in recent years. A multi-criterion comparative scoring of these synthesis approaches is presented in Figure 2a; BET surface areas by method are shown in Figure 2b; Figure 2c plots the response as a function of Pd loading, identifying the optimal window and the metallic-shorting regime; and Figure 2d summarises the key milestones timeline from 2017 to 2025.

### 2.1. Hydrothermal and Solvothermal Synthesis

Hydrothermal synthesis involves the controlled crystallisation of metal oxide nanostructures under autogenous pressure in an aqueous solution held at 120–220 °C. It remains one of the most widely used routes for producing SnO_2_ nanostructures because it is scalable and low in energy input, and it offers precise morphological control. Morphological tunability of SnO_2_ is achieved by altering solvent composition, pH, temperature, and capping-agent concentration. Yao et al. [1] formulated a glucose-assisted weak-acid hydrolysis protocol for SnCl_4_·5H_2_O that yields nanoporous SnO_2_. The mild acid conditions (pH 3–4, arising from glucose decomposition products) slow complete hydrolysis and prevent coalescence, yielding crystallites of ~10 nm average diameter that self-assemble into interstitial nanoporous networks. The resulting surface area reaches ~60 m^2^/g. Addition of PdCl_2_ enabled systematic variation of Pd loading (0.5–5.0 mol%); 2.5 mol% was identified as the optimum for CH_4_ response, maximising catalytic site density while avoiding coalescence of Pd islands that would reduce the metal–semiconductor interfacial area.

The morphological control over SnO_2_–ZnO and SnO_2_–Zn_2_SnO_4_ heterostructures can be attained by solvothermal variations with the mixed use of organic–aqueous solvents (e.g., ethanol–water and ethylene glycol–water). Despite not being pure systems, these hybrids arise in such architectures. They show the broad adaptability of the solvothermal platform; the same materials can, in fact, accept Pd decoration later. The aspect ratio of hydrothermally grown SnO_2_ nanorods, nanofibres, and other one-dimensional morphologies consists of a tunable SnCl_4_/H_2_O molar ratio [9]. Such a specific ratio leads to the synthesis of SnO_2_ nanorods and nanofibres from SnCl_4_·5H_2_O at 180 °C for 6 h.

### 2.2. Vapour–Liquid–Solid (VLS) Growth

The VLS mechanism has been employed in the field for producing single-crystal SnO_2_ NWs with the highest structural perfection available to the field. During a typical VLS process, a metallic catalyst (normally Au) is deposited on the substrate; at elevated temperature (700–900 °C) with a controlled Sn-bearing vapour (SnO or Sn powder oxidised in flowing gas) for vapour growth, that catalyst makes a eutectic alloy droplet that preferentially absorbs Sn species from the vapour phase. The phenomenon of supersaturation produces solid SnO_2_ nucleation and axial crystallisation beneath the droplet that produces single-crystal nanowires with a diameter in the range of 50–200 nm and length up to a few hundred micrometres. Pd decoration followed via UV photo-reduction: the VLS-grown SnO_2_ NWs were immersed in aqueous PdCl_2_ solution at ambient temperature and irradiated at 254 nm. This process reduced the Pd^2+^ species to Pd^0^ nanoparticles in situ. Well-dispersed Pd nano-particles of sizes ranging from 2 to 8 nm were obtained, and the density was controlled by the irradiation time.

Cai and Park [10] employed this VLS/UV-reduction method to synthesise Pd NP-decorated SnO_2_ nanowires, which led to a 12.7 times enhancement in hydrogen sensing response at the optimal Pd loading compared to the bare SnO_2_ NWs with a considerable improvement in selectivity against interfering gases. The present work and most of the high-performance Pd–SnO_2_ NW studies report H_2_ rather than CH_4_, which is an important finding. Both gas species activate Pd surface sites to give rise to reactive hydrogen species that migrate onto SnO_2_. However, CH_4_ possesses C–H bonds that require the successive abstraction of four σ-bonds during its utilisation (versus the dissociation of only a single H–H bond for H_2_, a molecule that possesses no C–H bonds). This suggests that the mechanistic transferability of the H_2_ data to CH_4_ performance is something of an approximation rather than a true equivalence. Metal oxide nanowires grown by VLS mechanisms using several oxides (SnO_2_, ZnO, and In_2_O_3_) have been studied in detail [11]. The generality of this method is confirmed to realise high-performance 1D sensing materials.

### 2.3. Electrospinning

Electrospinning offers a unique route to produce SnO_2_ nanofibres with very high surface areas (typically 100 to 200 m^2^/g) by calcination-induced combustion of polymer and Sn precursor in composite polymer–Sn jets. This is a typical process where SnCl_2_ or Sn(OAc)_2_ is dissolved in a polyvinylpyrrolidone (PVP)/ethanol solution and subjected to high-voltage electrostatic drawing to produce sub-micrometre composite fibres, which, upon calcination at 500–700 °C in air, collapse to hollow or solid SnO_2_ nanofibres. The findings of Bulemo et al. [12] reveal that electrospun hollow SnO_2_ microbelt sensitisation by apoferritin-templated Pt nanoparticles shows up to 7.8 times response enhancement at 2 ppm acetone and excellent stability over 25 sensing cycles at 90% RH. Note that Bulemo et al. employed Pt rather than Pd as the noble metal sensitiser; this study is referenced here as a structural analogue to illustrate the response benefits of hollow electrospun SnO_2_ morphology and not as a demonstration of Pd–SnO_2_ performance. The hollow shape and macroporosity of electrospun SnO_2_ fibres are advantageous for gas accessibility. Incorporation of Pd in electrospun SnO_2_ nanofibres typically involves adding Pd(OAc)_2_ or PdCl_2_ to the precursor solution, which yields embedded or surface-segregated Pd nanoparticles upon calcination.

### 2.4. Atomic Layer Deposition (ALD) and Physical Vapour Methods

ALD is the leading technology for conformal thin-film deposition and has been deployed to fabricate ultra-thin Pd films onto pre-formed SnO_2_ nanostructures. In ALD cycles, alternating pulses of a Pd precursor (e.g., Pd(hfac)_2_ or Pd(MeCp)Me_3_) and an oxidant or reductant (O_3_, H_2_O, or H_2_) achieve self-limiting monolayer deposition, typically in the 100–220 °C window [13], at growth rates of ~0.2 Å per cycle with sub-nanometre roughness. Thermal Pd(hfac)_2_/H_2_ processes operate at the lower end (≈100 °C), while ozone-based routes require 180–220 °C to form metallic rather than oxide films. Fang et al. [14] reported a Pd–SnO_2_/SiNW structure capable of detecting 1 ppm H_2_ at 300 °C (this is not room temperature but elevated temperature), response time of 9 s, and response value at 1.5% H_2_ of >9. The mechanism was ascribed to the Pd spillover, abundant SnO_2_ oxygen vacancies, and the Schottky barrier formed at the Pd/SnO_2_ interface. This work demonstrates that the incorporation of Pd-SnO_2_ active layers onto high-aspect-ratio 1D Si substrates is a possible route to CH_4_ sensing platforms. However, the 300 °C operating temperature does not meet the RT requirement that is the focal point of this review and should not be cited as proof of RT response.

A magnetron sputtering of Pd onto SnO_2_ nanostructures presents an intermediate approach; it is less conformal than ALD, but more uniform than wet chemical deposition. Control over the size (1–10 nm) and loading density of Pd clusters is possible through deposition time and power. The sensitive performance of the Pd/SnO_2_ nano-Schottky junction design by Song et al. [15], comprising Pd nanoclusters on SnO_2_ nanotube arrays, exhibited RT H_2_ sensing with a 1.6 ppb detection limit and stability for 100 days, triggering a paradigm shift due to the innovative milling of Pd/SnO_2_ junctions. The geometry of the 3D nanotube array endows it with a high density of Pd/SnO_2_ Schottky junctions per unit footprint, which is a key concept with an NW-based architecture.

### 2.5. Flame Spray Pyrolysis (FSP) and Photochemical Deposition

Flame spray pyrolysis allows for the continuous, high-speed production of Pd–SnO_2_ nanocomposite materials by burning liquid precursor sprays in a high temperature flame to give nanoparticle aerosols collected on filters. The ratio of the precursor concentrations (0.1–2 mol%) is used for controlling Pd loading, whilst rapid quenching from flame temperatures of >1500 °C can trap metastable alloy phases (PdSn) or lead to the formation of highly dispersed single-atom Pd sites. The FSP-derived Pd–SnO_2_ at 0.2 mol% Pd loading achieves sensor response enhancements of 2–6 times for acetone and CO compared to undecorated SnO_2_; at higher Pd loadings (1 mol%), enhancements of up to two orders of magnitude are reported [16]. This has been attributed to the very high Pd dispersion and intimate Pd–SnO_2_ contact achievable in the gas-phase process. According to the authors, FSP Pd–SnO_2_ mechanisms can be used in CH_4_ sensing contexts.

By irradiating SnO_2_ nanospheres or NWs in aqueous Pd^2+^ solutions with UV light, photochemical deposition enables us to selectively tune the oxidation state of Pd (metallic Pd^0^ versus PdO) and size of Pd particles by varying the irradiation parameters. The formation of PdSn alloys by the photochemical route is preferred because prolonged exposure to UV will allow reduction of the surface Sn along with deposition of Pd. According to Gschwend et al. [17], loading of Pd (0–3 mol%) on nanostructured SnO_2_ with [17] showed that photocapture this is achieved at loading of <0.2 mol%, acetone sensing (which is transferable to CH_4_) at 200–262 °C, which is a regime where catalytic oxidation conversion is ≈50%—measured response maximised before full oxidation cause a reduction gradient of the analyte in the gas phase which degrades the response. An optimum Pd loading should never be considered independently of the expected temperature of use. The key synthesis parameters, morphology outcomes, surface areas, and representative references for all discussed routes are consolidated in Table 1.

A direct comparison of the synthesis methods reveals clear practical trade-offs for CH_4_ sensor fabrication and mining deployment. Hydrothermal routes (Section 2.1) offer the most accessible scale-up pathway. Template-free hydrothermal synthesis of 1D SnO_2_ nanostructures has been systematically investigated, demonstrating control over aspect ratio, surface area, and crystallinity through precursor concentration and reaction temperature [18] (gram-scale batches, standard autoclaves, processing at 120–220 °C) but yield polycrystalline nanoporous assemblies rather than single-crystal NWs, limiting directional charge transport and the degree of Schottky junction engineering achievable [1]. VLS growth (Section 2.2) delivers the highest crystal perfection. Detailed morphological characterisation of VLS-grown crystalline SnO_2_ 1D nanostructures—including lattice parameter refinement, defect density mapping, and aspect-ratio quantification—has established reference benchmarks for assessing synthesis-route quality [19] and optimal 1D geometry for charge transport but requires high-temperature furnaces (700–900 °C) and yields milligram quantities on substrates, presenting a significant scalability bottleneck for mass production [10]. Electrospinning (Section 2.3) bridges scalability and surface area (>100 m^2^/g, large-area flexible substrates), but the resulting nanofibres are polycrystalline rather than single-crystal, and Pd nanoparticle homogeneity during co-calcination requires careful optimisation to avoid agglomeration [12]. ALD (Section 2.4) provides unmatched sub-nanometre Pd loading uniformity and is the preferred route for Schottky junction density engineering, but it is capital-intensive, slow (0.2 Å/cycle), and best deployed as a Pd post-decoration step on pre-formed VLS NWs rather than as the primary morphology route [13,14]. FSP (Section 2.5) is the only method demonstrated at continuous kilogram-scale production, making it industrially attractive, but the rapid quenching at >1500 °C generates mixed PdO/Pd° phases that require a controlled post-annealing step to optimise catalytic state before sensing [16]. For laboratory research targeting performance optimisation, a two-step VLS + ALD strategy currently offers the best compromise of crystal quality, junction uniformity, and Pd loading precision. For pilot-scale and industrial production, the FSP or hydrothermal + photochemical decoration routes are the more viable pathways.

## 3. Catalytic and Electronic Sensing Mechanisms

**Figure 3:**
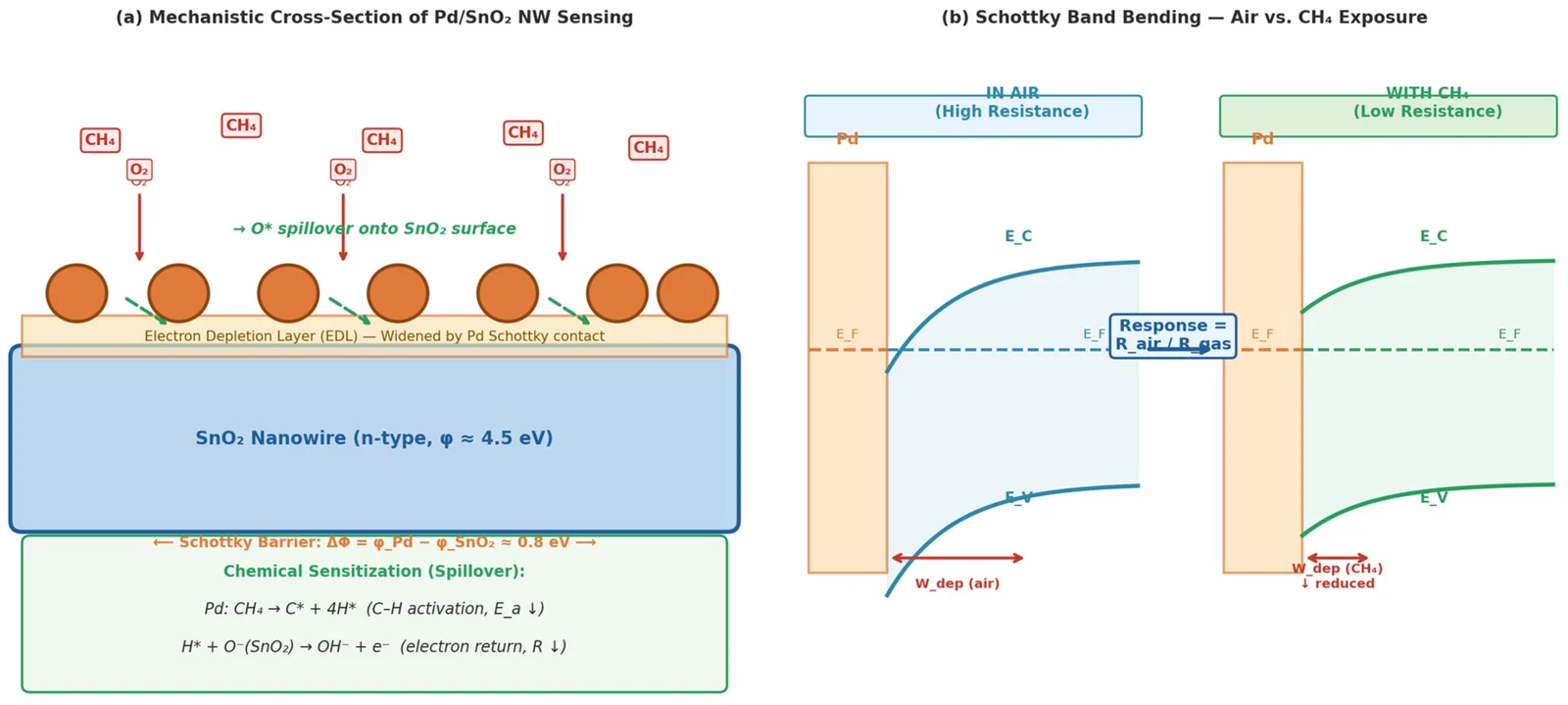
(**a**) Cross-sectional schematic of O*/H* spillover and Schottky sensitisation in a Pd–SnO_2_ nanowire. Blue arrows indicate the direction of O* spillover from Pd nanoparticles to the SnO_2_ surface; orange arrows show CH_4_ dissociation products (H*, CH_3_*) migrating onto the SnO_2_ surface; dotted lines mark the Schottky depletion layer boundary; colours indicate energy levels. (**b**) Energy band diagrams for the air (high-resistance) state (solid lines) versus the CH_4_-exposure reduced-depletion, low-resistance state (dashed lines). The shaded region indicates the space-charge depletion layer (W_d).

The dual sensitisation mechanism is illustrated schematically in Figure 3a; the corresponding band diagrams for the sensor in air (high-resistance state) and upon CH_4_ exposure (reduced depletion, low-resistance state) are shown in Figure 3b. Un-doped *n*-type SnO_2_ responds to its gas environment through a well-established surface chemisorption sequence. O_2_ at atmospheric concentrations adsorbs onto surface sites of SnO_2_ and captures conduction-band electrons (e^−^) to create anion species. The predominant form of the adsorbed oxygen anion species is temperature-dependent: O_2_^−^ (T < 150 °C), O^−^ (150 < T < 400 °C), O^2−^ (T > 400 °C). The withdrawal of electrons from the surface layer creates an R-space charge region that increases sensor resistance in air (R-air). The introduction of CH_4_ leads to its high-temperature reaction with the oxygen surface species given by CH_4_ + 4O^−^ → CO_2_ + 2H_2_O + 4e^−^, whereby electrons are returned to the conduction band, and this reduces R_gas. The sensing response of R_air/R_gas is given for reducing gases on *n*-type semiconductors. The main limitation that pristine SnO_2_ has for CH_4_ is specifically that the strong C–H bond (439 kJ mol^−1^) of CH_4_ would require a temperature above 200 °C for thermally activated surface oxidation, which can be uniquely addressed by Pd catalysis.

### 3.1. Chemical Sensitisation: The Pd Spillover Effect

The spillover effect refers to the process whereby atoms or molecules that have been adsorbed on a catalytically active metal (the ‘donor’) diffuse from there to an oxide support that is adjacent (the ‘acceptor’), where they take part in surface reactions. These surface reactions would not be kinetically accessible on the bare support. Pd nanoparticles are dissociation centres of O_2_ and CH_4_ for Pd-SnO_2_ CH_4_ sensing. At ambient conditions, Pd breaks down O_2_ into highly reactive atomic oxygen (O*) species that migrate onto SnO_2_ surface sites, enhancing and at times replacing the less efficient thermally activated O_2_ chemisorption on bare SnO_2_. The mechanism of oxygen activation facilitated by Pd gives rise to a markedly larger density of reactive oxygen species on the surface of SnO_2_ at room temperature (RT); this preconditioning improves the phenomenally fast response of the sensor to analytes.

When CH_4_ is present, Pd can mediate C–H bond activation at the same time: surface Pd dissociates CH_4_ into H* and CH_3_* radicals at much lower temperatures than those needed on SnO_2_ alone, facilitated by the ability of the Pd d-band to overlap with CH_4_ anti-bonding orbitals. The species spill onto SnO_2_, consuming oxygen surface species and leading to electron release. Direct mechanistic evidence for spillover in a closely related system was provided by Cai et al. [9] (note: H_2_ analogue; CH_4_ spillover is inferred by analogy), who used ex situ XPS, in situ Raman spectroscopy, and DFT on a hollow Pd-NiO/SnO_2_ nanocavity to confirm that Pd–H bond formation and subsequent hydrogen dissociation onto the oxide surface are the rate-determining steps. The distinctive nanocavity geometry effectively contains H_2_ molecules, maximising spillover contact time and achieving 100 ppb detection limits at 230 °C. The spillover sensitisation concept is generalisable across multiple reducing gases and material systems: CO detection via Pd/SnO_2_/porous g-C_3_N_4_ nanocomposites has confirmed that a conductive g-C_3_N_4_ network enhances electron mobility and catalytic spillover efficiency simultaneously [20]; Pd-confined spillover in porous Co_3_O_4_ hollow polyhedra produced fast, highly sensitive ethanol detection, confirming that Pd-mediated spillover extends to gases beyond CH_4_ [21]; and reverse oxygen spillover—where lattice oxygen migrates from the support to the noble metal—has been identified in Sn-doped Pt/TiO_2_, revealing the bidirectional nature of spillover at Pd-group metal–oxide interfaces [22]. Operando spectroscopic evidence for reversible H_2_ spillover between Pd nanoparticles and MOF frameworks has further established the mechanistic basis for Pd chemical sensitisation in nanoporous matrices [23]. Additionally, polyaniline/SnO_2_/Pd hybrid composites have demonstrated room-temperature H_2_ sensing responses of ~540% at 0.4% H_2_—among the highest reported for any Pd–SnO_2_-based RT system [24]—confirming that matrix engineering can dramatically amplify the spillover response. According to Oleksenko et al. [25], the ≤1000 ppm CO sensing response (relative to CH_4_) observed in sol–gel Pd/SnO_2_ nanomaterials arises because CO is catalytically more reactive than CH_4_ toward oxidation. Equivalent CH_4_ spillover-driven response amplitudes would therefore require higher Pd loading and/or elevated temperature.

The spillover efficiency relies heavily on the chemical state of palladium. The metallic form of palladium, denoted as Pd^0^, causes both O_2_ and CH_4_ to dissociate efficiently. The oxidised form of palladium, denoted as PdO, is the form that occurs under oxygen-rich conditions at high temperatures. C–H activation has different kinetics with PdO. Yue et al. [26] showed by operando TEM and NAP-XPS that catalytically active Pd nanoparticles exhibit dynamic Pd/PdO phase coexistence during methane oxidation. The strained PdO phase at the edge of the nanoparticle was identified as an especially reactive site for C–H bond activation. The applicability of oscillatory behaviour or redox equilibrium in phase inter-conversions will vary with the reaction conditions and remains a subject of research [27]. The research indicates that the optimal operating conditions for sensors to detect methane include an air atmosphere with moderate temperature. When ventilated in air at a moderate temperature, the PdO/Pd surface phase stabilisation would yield a stable sensor performance.

### 3.2. Electronic Sensitisation: Schottky Barrier Modulation

The contact of Pd metal (φ_Pd ≈ 5.1–5.6 eV, depending on surface orientation) with *n*-type SnO_2_ (φ_SnO_2_ ≈ 4.5 eV, electron affinity χ ≈ 4.0 eV) forms a Schottky barrier at the Pd/SnO_2_ interface. Upon contact, electrons flow from SnO_2_ to Pd until Fermi-level alignment is achieved, creating a depletion region on the SnO_2_ side whose width W_d is proportional to (2εV_bi_/eN_d_)^(1/2)^. Here, ε is the permittivity of SnO_2_; V_bi_ is the built-in potential; e is the electron charge; and N_d_ is the donor density. In SnO_2_ NWs, this depletion caused by Pd addition participates in the already present depletion layer induced by surface oxygen chemisorption in air. This results in an extraordinary baseline resistance R^air^, which considerably enhances the relative resistance change (R^air^/R^gas^) during CH_4_ exposure.

According to DFT calculations conducted by Xue et al. [3] on Pt-Pd/SnO_2_ mesoporous systems, the observed orbital hybridisation of Pd d-states with CH_4_ molecular orbitals is induced by covalent bonding interactions between the bimetal nanoalloys and the SnO_2_ support, as well as rapid electron transfer from the Pt-Pd nanoalloys to the SnO_2_ support. These calculations help quantify the electronic sensitisation pathway at the atomic level. The best loading of Pd for electronic sensitisation is around 1.0–2.5 mol% (or ~1–3 nm Pd clusters at an estimated surface coverage of which is ~10 Pd atoms/nm^2^, using typical packing densities of Pd nanoparticles on metal oxide supports): below this limit, there are insufficient Schottky junctions formed; above this limit, Pd islands form a continuous conductive network, ‘shorting’ the SnO_2_ channel and lowering sensitivity (the ‘metallic shorting’ effect). Song et al. [15] engineered nano-Schottky junctions in Pd/SnO_2_ nanotube arrays, achieving 1.6 ppb H_2_ detection and 100-day stability. Their work explicitly demonstrated that junction engineering, rather than simply maximising Pd loading, is the decisive design principle.

### 3.3. Synergistic Effects and Hybrid Nanoarchitectures

In reality, chemical and electronic sensitisation are not independent; the Schottky barrier induced by Pd and the Pd-mediated surface oxygen density are mutually reinforcing. Increased spillover-generated reactive oxygen species density on SnO_2_ can enhance baseline resistance (more profoundly depleted) while also supplying more reaction partners to H* species derived from CH_4_. The Schottky barrier amplifies the change in resistance that occurs when those reaction partners are consumed. Optimal synergy is achieved at Pd loadings of 1.0–2.5 mol%, where both effects are maximised without inducing metallic shorting.

The addition of rGO or CNT networks to Pd–SnO_2_ composites opens up a third sensitisation route, as the high conductivity and oxygen functionality density of rGO provides RT-operating electron transport channels and oxygen adsorption sites. Xia et al. [2] showed that the palladium nanoparticle (Pd NP)-decorated ZnO/reduced graphene oxide (rGO) hybrids under 470 nm visible light illumination displayed 6.3–63.4% CH4 detectable 25–10,000 ppm RT response (note: ZnO matrix, not SnO_2_; included as a mechanistic analogue for rGO contribution). The visible-light photocatalytic activation of Pd generates additional photogenerated electron–hole pairs that help to activate oxygen at the Pd sites. This has been reported to be a transferable mechanism to respond to Pd–SnO_2_/rGO composites. Thus, direct experimental study validates these claims but often does not extend to the SnO_2_ NW/rGO systems directly. According to Zhang et al. [28], Pd-doped rGO/ZnO-SnO_2_ nanocomposites gave a 9.4 response at 100 ppm H_2_ and a 50 ppb detection limit at 380 °C. rGO enhances both sensitivity and speed, but the high operating temperature highlights how rGO by itself does not lead to RT performance. Studies on the behaviour of multi-walled CNT–SnO_2_–Pd composites show that the networks formed by CNTs act to create high-conductivity percolation pathways plus sensitisation by the chemical nature of CNTs themselves. This results in a strong enhancement effect when compared to pure SnO_2_ for relevant industrial gases. On the downside, dedicated studies on SnO_2_/CNT/Pd CH_4_ and similar sensing remain scarce, and the enhancement to be expected is system-dependent. The mechanistic contributions of chemical and electronic sensitisation, and their synergistic combination, are compared in Table 2.

## 4. Performance Metrics and Benchmarking

**Figure 4:**
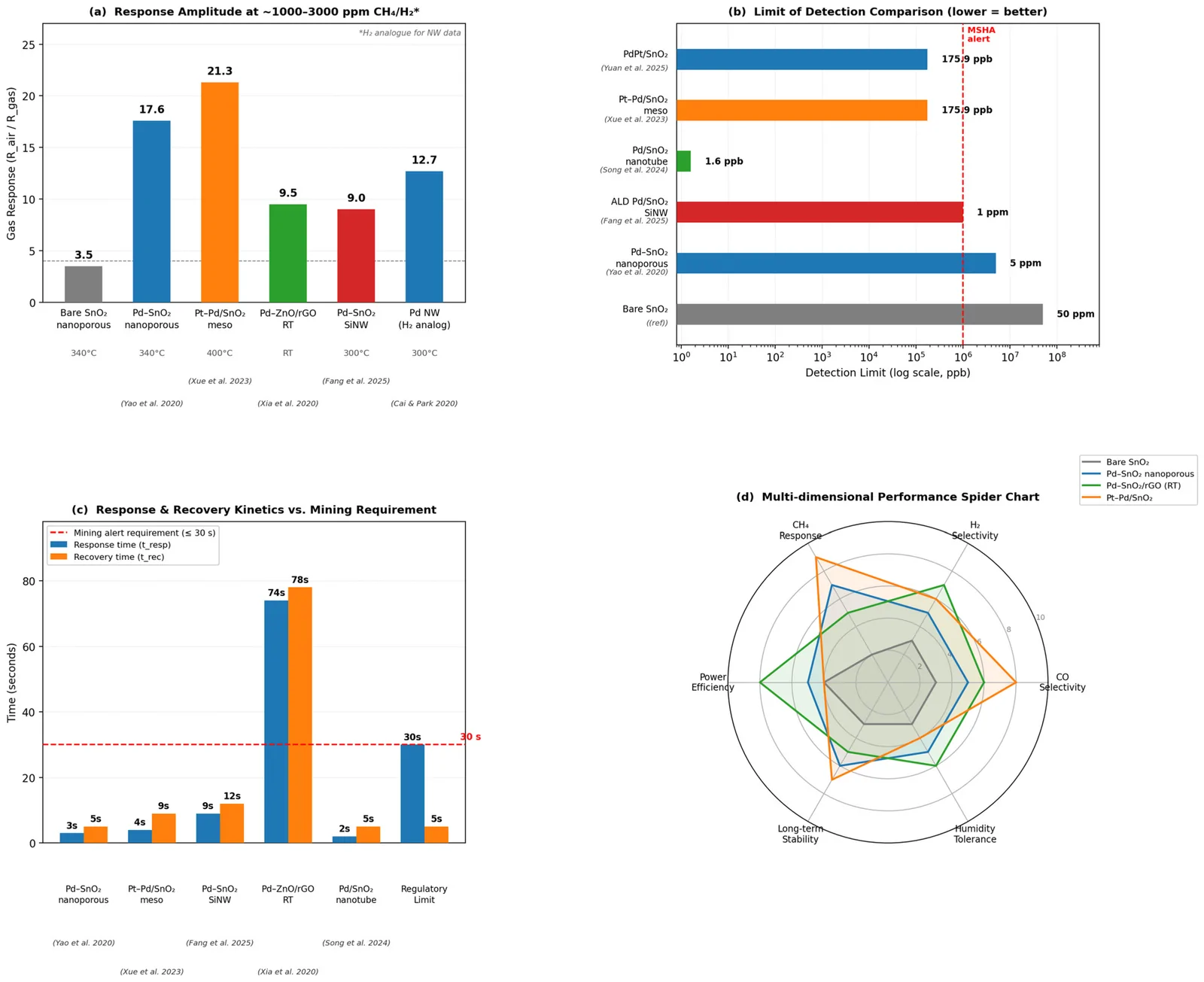
(**a**) Response amplitude. (**b**) LOD log–scale comparison. (**c**) Kinetics vs. 30 s alarm limit. (**d**) Multi-criteria spider chart across six performance dimensions. Data: refs [1,2,3,10,14,15,30].

Quantitative performance benchmarks for the reviewed sensor systems are presented in Figure 4: response amplitudes are compared in Figure 4a, LOD values on a log scale in Figure 4b, kinetics against the 30 s MSHA alarm limit in Figure 4c, and a multi-criteria spider chart across six performance dimensions in Figure 4d. It is difficult to systematically compare the sensor performance in the literature of Pd–SnO_2_ because the responsivity definitions, analyte concentrations, operating conditions, and substrate geometries are non-standardised. This review uses R_air/R_gas (R_air is the resistance in air and R_gas is the resistance in a gas of interest) as the main response metric reported for *n*-type reducing gas sensors, in agreement with most of the literature adopted in this review, and percentage-change responses are also noted separately if reported. Table 3 provides important performance values obtained from the main scientific literature identified using a systematic database search from 2019 to 2025. This text says that when data is taken by H_2_ sensing studies or non-NW nanostructures, it is mentioned explicitly; that is, the case data may not be seen as directly predictive of RT CH_4_ NW performance.

**Table 3:** Comparative performance metrics for representative Pd–SnO_2_ and analogous noble metal–SnO_2_ sensor systems for CH_4_ and H_2_ detection. H_2_ data (*) are included as mechanistically analogous cases where direct RT CH_4_ NW data are absent. Non-NW morphologies and Pt-containing systems are explicitly noted; their inclusion is as structural or mechanistic analogues only and does not imply equivalence with pure Pd–SnO_2_ NW performance.

Material/System	Morphology	Op. Temp (°C)	Response (Conc.)	t_resp/t_rec (s)	LOD	Reference
2.5 mol% Pd–SnO_2_	Nanoporous NPs	340	17.60 @ 300 ppm CH_4_	3/5	–	Yao et al., 2020 [1]
Pd–SnO_2_/rGO	Composite film	RT (25)	9.5% @ 12,000 ppm	Fast/Fast	Low ppm	Nasresfahani et al., 2017 [29]
Pd–SnO_2_ nanofilm/SiNWs	1D hybrid film	300	>9 @ 1.5% H_2_ *	9/–	1 ppm	Fang et al., 2025 [14]
Pd NP-dec. SnO_2_ NWs	VLS NWs	300	12.7× vs. bare (H_2_) *	Enhanced	ppb-level	Cai et al., 2020 [10]
Pt-Pd/SnO_2_ mesospheres	Mesoporous spheres	400	21.33 @ 3000 ppm CH_4_	4/9	175.9 ppb	Xue et al., 2023 [3]
Pd–ZnO/rGO (vis. light)	Composite	RT	6.3–63.4% @ 25–1% CH_4_	74/78	Low ppm	Xia et al., 2020 [2]
Pd-NiO/SnO_2_ nanocavity	Yolk-shell hybrid	230	Ultra-high @ H_2_ *	–	100 ppb	Cai et al., 2023 [9]
Pd–rGO/ZnO-SnO_2_	Nanocomposite	380	9.4 @ 100 ppm H_2_ *	4/8	50 ppb	Zhang et al., 2022 [28]

Note: H_2_ data (*) included as mechanistically transferable; all CH_4_ data are direct measurements. LOD = limit of detection; RT = room temperature (25 °C); t_resp/t_rec = response time/recovery time to 90% of steady-state signal; NPs = nanoparticles; NWs = nanowires; SiNWs = silicon nanowires; rGO = reduced graphene oxide; vis. light = visible-light-activated photocatalysis.

### 4.1. Response Amplitude

The response of pure SnO_2_ sensors to CH_4_ at 500–3000 ppm and 200–400 °C is typically around 2–10. Pd decoration consistently improves responses by 3–100 times. A response of 17.60 at 300 ppm CH_4_ and 340 °C was reported for 2.5 mol% Pd–SnO_2_ in a landmark study by Yao et al. [1] employed a nanoporous nanoparticle morphology rather than a nanowire architecure; this result should therefore not be classified under Pd–SnO_2_ NW performance. The Pt-Pd/SnO_2_ mesoporous system of Xue et al. [3], which delivered 21.33 at 3000 ppm CH_4_ at 400 °C, employs a bimetallic PtPd formulation in a mesoporous sphere geometry, neither pure Pd nor NW. The range of 17.6–21.3 cited in this review thus constitutes the broad performance of Pd-containing SnO_2_ nanostructures, rather than NW-specific values. The literature records no dedicated NW performance data for CH_4_, which is a main research gap discussed in Section 6.1. RT composites that incorporate rGO attain 9.5% response to 12,000 ppm CH_4_ at 25 °C (as shown in Nasresfahani et al. [29], a precursor study from outside the 2020–2026 primary scope cited here as a foundational RT baseline), while visible-light-assisted non-NW morphologies, including WO_3_-decorated flower-like SnO_2_ [31] and Au-nanoparticle-decorated SnO_2_ nanoflowers [32] have further been reported to enhance CH_4_ sensing response through additional active surface sites and spillover-assisted oxidation. Pd–ZnO/rGO attains 63.4% at 1% CH A self-assembled mulberry-like ZnO/SnO_2_ hierarchical structure has also achieved ~56.1% response to 2000 ppm CH_4_ at room temperature under 55% relative humidity [33], confirming that interfacial strain-enhanced piezoelectric polarisation is an effective RT sensitisation route distinct from Pd decoration at RT [2]. Single-crystal Pd–SnO_2_ NW systems outperform bare SnO_2_ NWs by 12.7 times for H_2_—the closest mechanistic analogue, while nano-Schottky junction engineered Pd/SnO_2_ nanotube arrays produced 1.6 ppb H_2_ at room temperature [15].

### 4.2. Response and Recovery Kinetics

The time taken for 90% of the sensor output signal to reach the steady state (t_90) is a critical parameter for mining safety applications. The regulations require an alarm to activate within 30 s of the start of a hazardous concentration. According to Yao et al. [1], a response/recovery time of 3/5 s has been reported for nanoporous Pd–SnO_2_ at 340 °C and 300 ppm CH_4_. This is attributed to the open nanoporous structure, which serves to enable fast gas diffusion combined with Pd catalysis. Fang et al. [14] reported a 9 s response time for 1.5% H_2_ at 300 °C on Pd–SnO_2_/SiNW architectures, while Pt-Pd/SnO_2_ mesospheres showed 4 s/9 s at 3000 ppm CH_4_ [3]. Visible-light-activated room-temperature systems show longer kinetics (74 s/78 s for Pd–ZnO/rGO [2]) owing to the photocatalytic O_2_ activation being rate-limiting. This response time is acceptable for continuous ambient monitoring but does not satisfy the <30 s MSHA alarm requirement. MEMS-based low-power catalytic sensors are designed for mining and operate with a 95% reduction in total power consumption when compared to traditional catalytic bead sensors in pulsed operation [34]. The same reduction strategy is also applicable to Pd–SnO_2_ NW MEMS devices.

### 4.3. Limit of Detection (LOD)

The response of Pd–SnO_2_ sensors spans low-ppm in early hydrothermal systems to sub-ppb in deliberately engineered nanoarchitectures. ALD Pd–SnO_2_/SiNW sensors detect H_2_ at 1 ppm at 300 °C [14], while nano-Schottky junction Pd/SnO_2_ nanotube arrays achieve a state-of-the-art H_2_ LOD of 1.6 ppb at RT [15]. These results represent the current benchmark for H_2_ sensing in Pd–SnO_2_ architectures. The lowest CH_4_ LOD currently reported is 175.9 ppb, achieved by the Pd_x_Pt/SnO_2_ mesoporous system operating at 400 °C [30]. Two-dimensional SnO_2_ disk morphologies have demonstrated H_2_ sensing with a response of 15.5 at 100 ppm and an LOD of 1 ppm at 200 °C [35], illustrating how morphology dimensionality governs surface area and consequently detection limit. Consequently, the Pd–SnO_2_ NW is not the sole source of the Pd_x_Pt/SnO_2_ system, which has the implication that the great sensitivity is a synergistic contribution of Pt co-decoration and mesoporous, not NW morphology. To date, no published work demonstrates a comparable CH_4_ LOD from a pure Pd–SnO_2_ NW system at RT. This is the main issue.

### 4.4. Selectivity and Humidity Tolerance

Selectivity improvements in Pd–SnO_2_ systems are strongly condition-dependent and must be interpreted in the context of the specific interfering gas mixture, operating temperature, and material architecture. At room temperature, Pd-sensitised nanocrystalline SnO_2_ thin films demonstrated high discrimination of CH_4_ against NH_3_, H_2_S, NO_2_, and NO, while CO selectivity was achieved at 100 °C [36]. At elevated temperatures, bimetallic Pd–Au–SnO_2_ nanosheets enable temperature-switchable dual-analyte selectivity—CH_4_ at 250 °C and CO at 110 °C [37]—demonstrating that operating temperature can itself serve as a selectivity tuning parameter. For complex underground mine atmospheres containing simultaneous CH_4_, CO, H_2_, H_2_S, and CO_2_, a machine learning-augmented sensor array achieved 91.6% blind classification accuracy [38], representing the most operationally realistic selectivity demonstration in the reviewed literature. These findings collectively indicate that no single material configuration achieves universal selectivity; rather, selectivity must be engineered through a combination of bimetallic decoration, operating temperature modulation, and signal-processing strategies.

The performance of Pd–SnO_2_ sensors needs to be selective enough to work in coal mines, which contain not only CH_4_ but other gases as well, like CO (10–100 ppm), CO_2_ (up to 1%), H_2_ (up to 100 ppm), H_2_S (trace), and water vapour (50–90% RH). If measuring by similar concentrations, the pristine SnO_2_ has a very low (less than 50%) selectivity for CH_4_ with regard to H_2_ and CO. Pd decoration enhances selectivity through its stronger catalytic affinity toward CH_4_ relative to CO at room temperature. Gangwar et al. [36] confirmed that room-temperature-sputtered Pd-sensitised nanocrystalline SnO_2_ thin films have achieved a ~94.5% response to 91 ppm CO at 100 °C with high selectivity over NH_3_, H_2_S, NO_2_, and NO [36], confirming that Pd sensitisation promotes analyte discrimination at sub-200 °C than CO at room temperature. This occurs in the reverse of the high-temperature behaviour. However, some residual H_2_ cross-sensitivity remains. The use of metal alloy composition to promote sensor metal selectivity, which is hardware-unmodified, has been illustrated with bimetallic PdAu decoration of SnO_2_ nanosheets, enabling temperature-switchable dual selectivity (acetone at 250 °C, formaldehyde at 110 °C) [37]. It should be noted that Li et al. [37] validated PdAu bimetallic decoration specifically for acetone and formaldehyde selectivity under temperature-switching conditions; no experimental data in that study demonstrate improved selectivity toward CH_4_, the primary analyte of this review. The applicability of PdAu decoration for CH_4_-selective detection, therefore, remains an open research question and is identified as a priority direction for future work (Section 6.2). Recent investigations into SnO_2_ heterostructure systems have further demonstrated that interfacial engineering significantly modulates both sensitivity and selectivity in the presence of interfering gases and humidity [39]. The PdPt–SnO_2_ systems are designed to prevent cross-sensitivity at the sensor surface [3,30]. This is achieved via the combination of low-temperature oxophilicity and high-temperature catalytic activity. Optimised Pd-decorated SnO_2_ sensors have furthermore been validated for dissolved CO detection in transformer insulating oil at room temperature, with a LOD of ~13.3 ppm dissolved CO, extending their applicability to industrial transformer fault monitoring [40]. SnO_2_ exhibits significantly enhanced selectivity.

The response of bare SnO_2_ sensors is reduced by 20–50% in the presence of humidity [41,42]. Pd-capped SnO_2_ thin films with optimised Pd surface coverage have demonstrated improved humidity tolerance in H_2_ detection without sacrificing sensitivity [43], suggesting that Pd density engineering is a practical pathway for humidity-resilient CH_4_ sensors. This is because they will compete with hydrogen that comes from CH_4_ for surface oxygen sites. Also, the humidity will induce surface hydroxylation and thereby impact the surface chemistry. Research suggests three strategies to reduce humidity drift in various sensor architectures. The first is to add a core–shell CeO_2_/SnO_2_ nanostructured sensor with an OH-resistant CeO_2_ outer shell but a SnO_2_-sensing core. The second is organosilane (hydrophobic) surface functionalisation. The third is operation at elevated temperature (T > 200 °C), where water desorption kinetics then increase above adsorption kinetics. When it comes to RT sensors, operating with UV-assistance has been shown to continuously regenerate surface-active sites. Separately, optimisation of CeO_2_-SnO_2_ binary oxide microstructures for methane combustion catalysis has confirmed that CeO_2_ enrichment provides OH-resistant stability and improves Pd redispersion under wet conditions [44], supporting its use as an outer shell in core–shell humidity-mitigation architectures [2].

## 5. Mining Safety Applications

**Figure 5:**
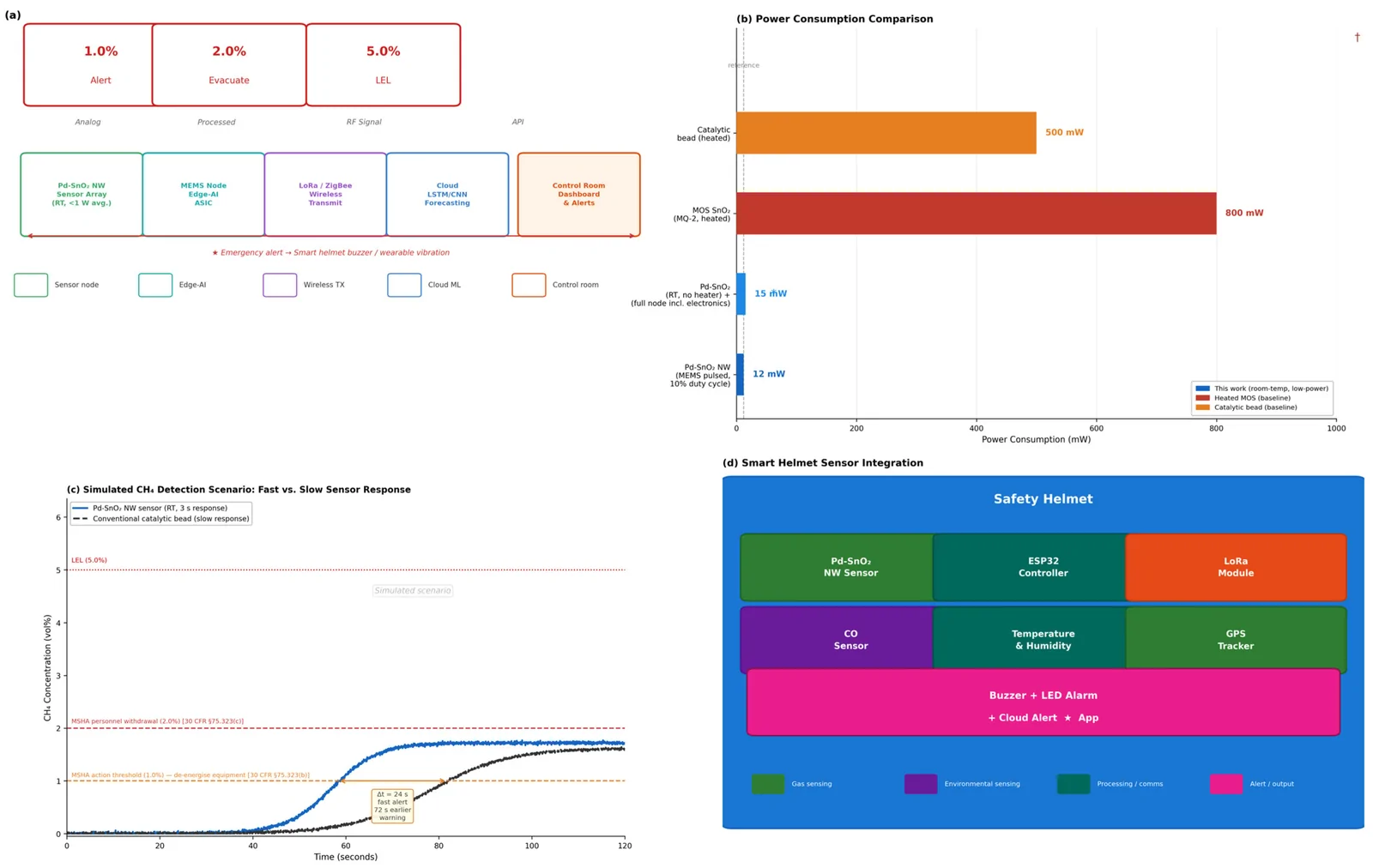
(**a**) End-to-end IoT architecture. (**b**) Power consumption comparison. (**c**) Simulated CH_4_ detection alarm scenario. (**d**) Smart safety helmet sensor integration schematic.

The end-to-end IoT deployment architecture for Pd complementary optical approaches—notably, tunable diode laser absorption spectroscopy (TDLAS)—achieves sub-ppm CH_4_ detection but requires bulky, power-intensive components incompatible with wearable underground deployment [45]; MOS-based IoT architectures are therefore the more practical near-term solution, and their substantially higher capital cost and maintenance requirements relative to solid-state chemiresistive sensors further reinforce the economic case for Pd–SnO_2_ NW development. SnO_2_ NW sensors are depicted in Figure 5a; power consumption benchmarks are shown in Figure 5b; a simulated CH_4_ detection alarm scenario is provided in Figure 5c; and Figure 5d illustrates smart safety helmet sensor integration. The Pd–SnO_2_ NW sensor technology must become a deployable underground mining safety device that satisfies a multi-dimensional specification matrix involving regulatory, intrinsic safety, environmental, and communication compatibility specifications, as opposed to merely a laboratory proof of concept. Experts are already engaged in the certification of more advanced sensors that will not only make the mining process safer but also more efficient.

### 5.1. Regulatory Compliance Framework

Monitoring of gas in underground coal mines is governed by national regulatory regimes that specify action threshold concentrations. In the United States, MSHA (30 CFR Part 75, §75.323) requires de-energisation of electrical equipment and ventilation adjustments at 1.0% CH_4_, withdrawal of personnel from working places at 1.5%, and immediate evacuation of all personnel at 2.0% CH_4_ (imminent danger level). In the European Union, mine environments are classified as Zone 1 (gas present occasionally) or Zone 0 (gas present continuously) under ATEX Directive 2014/34/EU, and sensor equipment must carry ATEX Category 1G or 2G certification. ATEX-certified equipment must have an external housing surface temperature below 80% of the autoignition temperature of the most easily ignited gas present—for CH_4_ (autoignition 537 °C), this equates to a housing surface temperature < 430 °C. Note that this restriction applies to the external housing rather than the sensing element and is most constraining for designs that rely on a heater. RT Pd–SnO_2_ NW sensors eliminate the heater element entirely and are therefore compliant by design. China’s Coal Mine Safety Regulations (Table 19) sets alert and alarm thresholds at 0.5% and 1.0% CH_4_ respectively (readers are directed to the primary regulatory text for definitive values). Because RT Pd–SnO_2_ NW sensors operate without heater elements, they satisfy the surface-temperature requirement intrinsically and eliminate the heater as a potential ignition source, conferring an inherent safety advantage over heated MOS architectures.

### 5.2. IoT Integration and Smart Monitoring Architectures

More distribution and dynamic underground mine monitoring is made possible through the integration of low-power gas detecting and communication infrastructure to the Internet of Things (IoT). Monitoring architectures are shifting away from fixed-point installations toward dynamic wireless sensor networks distributed across the working face. A wearable smart helmet incorporating a ZigBee-based wireless monitoring system has been demonstrated for real-time underground gas detection [46], monitoring CH_4_, CO, temperature and humidity successfully. The underground operation is uniquely suited to low-power, long-range, through-rock 868 MHz signals from a LoRa transceiver. A smart underground monitoring node containing a LoRa transceiver, a low-power and low-cost PID sensor, and a temperature and humidity sensor was implemented in a working coal mine (Pandaveswar Colliery, Eastern Coalfields Limited, Paschim Bardhaman, West Bengal, India). Cloud-based dashboards hold the data of CH_4_, CO, T and H [47]. An IIoT (Industrial Internet of Things) device incorporating LSTM-based multivariate methane forecasting has been demonstrated with 4.23% MAPE in field deployment [38].

Smart helmet integration represents the most human-centred deployment paradigm for Pd–SnO_2_ NW methane sensors, positioning the sensing node directly at the point of potential exposure.

Research prototypes have been developed for MQ4 (CH_4_) and MQ7 (CO) with ESP32 Controllers, which relay their readings onto the ThingSpeak Cloud platform over local Wi-Fi and with on-board buzzer alarms and app interfacing [48]. Commercially available catalytic bead (MQ-series) sensors are currently used in smart helmets; while being drop-in replacements, Pd–SnO_2_ NW MEMS sensors use sub-mW power and a chip-scale footprint of <1 cm^2^. Moreover, deploying these MEMS sensors would allow for extended battery life by several orders of magnitude, making always-on continuous monitoring feasible with no duty-cycle compromises whatsoever. MEMS-based wireless methane sensors operating in pulsed mode have been confirmed to consume only 2.4% of the power used by traditional catalytic sensing elements [34]. This confirms the feasibility of battery-powered RT Pd–SnO_2_ sensor nodes.

### 5.3. Artificial Intelligence and Machine Learning Integration

Sensor arrays used in conjunction with ML (machine learning) methods have been engineered to overcome the selectivity limitations of individual sensors [38]. When a multi-element (Pd–SnO_2_/Pd–ZnO/Pd–In_2_O_3_) sensor array was exposed to a training gas mixtures containing constituents of the mining atmosphere (CH_4_, CO, H_2_, H_2_S, CO_2_ and water vapour) the pattern recognition algorithms (principal component analysis (PCA), support vector machines (SVM), convolutional neural networks (CNN; SqueezeNet)) were able to not only classify the identity of the gases present but also estimate the concentration of the gases. Reported accuracies of up to 91.6 so far in blind testing with simulated mine gas mixture concentrations [38]. ML approaches relax selectivity requirements on individual sensors. The array-plus-ML sensor strategy tolerates known cross-sensitivities. In other words, it does not require perfect single-component selectivity from individual sensors. As long as the cross-sensitivity patterns are discriminable and not confusing, the sensor array-plus-ML approach can be successful. This has deep consequences for the deployment of Pd–SnO_2_ NW sensors: it allows for the acceptance of a known H_2_ cross-sensitivity. The current status of Pd–SnO_2_ NW technology against key mining safety requirements is summarised in Table 4.

## 6. Challenges, Gaps, and Future Perspectives

**Figure 6:**
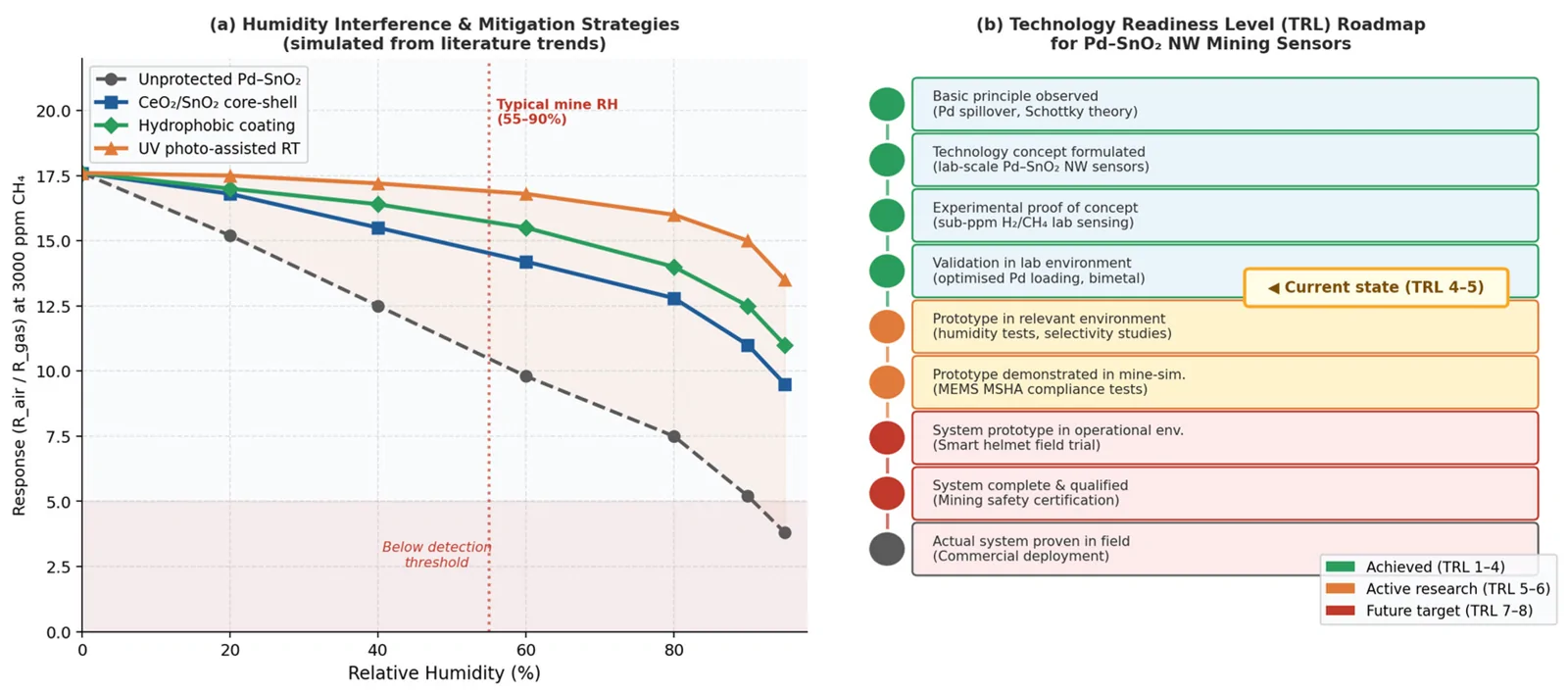
(**a**) Humidity interference and mitigation strategies. (**b**) TRL roadmap from basic research (TRL 1) to commercial mining deployment (TRL 9); current status ~TRL 4–5.

Humidity interference mechanisms and mitigation strategies are summarised in Figure 6a. The technology readiness level (TRL) roadmap, indicating the current status of Pd–SnO_2_ NW systems at approximately TRL 4–5 and the pathway to certified mining deployment at TRL 9, is shown in Figure 6b.

### 6.1. Literature Gaps

Palladium-decorated tin oxide nanowires have been investigated by several groups for room-temperature methane sensing; however, no quantitative direct demonstration of a competitive pure Pd–SnO_2_ NW device (response > 5 at 1000 ppm CH_4_ and 25 °C, t_90_ < 30 s, LOD < 50 ppm) has yet been published. Most high-performance RT CH_4_ reports instead employ non-NW morphologies—nanoporous assemblies, thin films, and rGO composites (see references in Section 1)—or rely on photo-activation. Conversely, studies on high-performing Pd–SnO_2_ NWs typically use H_2_ as the primary analyte and treat CH_4_ as a secondary or additional target. This reflects a real thermodynamic barrier: the C–H bond in CH_4_ (439 kJ mol^−1^) compared with the H–H bond of H_2_ (≈436 kJ mol^−1^) explains why RT CH_4_ activation is mechanistically more demanding, and H_2_ therefore serves as a more tractable model analyte even in systems intended for CH_4_ deployment.

Data on the long-term operational stability is another gap. The published reports on the stability of Pd–SnO_2_ were mainly in laboratory conditions over a 30-day to 90-day duration, while mining sensor certification typically requires 6–12-month field stability with <10% drift. The effects of Pd sintering due to thermal cycling—caused by Pd sulphide poisoning from H_2_S found in mine atmospheres—and gradual SnO_2_ surface hydroxylation in high humidity conditions still need to be characterised over timescales that are relevant in operating conditions.

### 6.2. Bimetallic and Alloy Catalysts

A notable and conducive route to overcoming the selectivity and stability challenges at the same time in the near term is bimetallic PdPt and PdAu nanoalloy decoration of SnO_2_ nanostructures. PdPt alloys leverage the C–H activation activity of Pd alongside the oxophilicity of Pt to preferentially oxidise CO—which otherwise poisons bare Pd at room temperature—thereby enhancing catalytic turnover for CH4. Xue and colleagues [3] based on its improved CH4 response (21.33 at 3000 ppm), Pt-Pd/SnO_2_ mesoporous spheres have better CH4 response (21.33 at 3000 ppm) compared to single-metal Pd or Pt, attributing synergy to orbital hybridisation effects confirmed by DFT (density functional theory). As shown by Yuan et al. [30], PdxPt/SnO_2_ sensors working across ultra-wide CH_4_ concentration (50–20,000 ppm) with CH4 LOD = 175.9 ppb prove rational bimetal alloy engineering access to the ppb regime. Li et al. [37] validated PdAu bimetallic decoration of SnO_2_ nanosheets, demonstrating temperature-switchable dual selectivity for acetone (250 °C) and formaldehyde (110 °C), thereby confirming the viability of alloy engineering for targeted analyte discrimination. However, it is important to emphasise that Li et al. [37] validated PdAu selectivity only for acetone and formaldehyde; targeted investigation of PdAu–SnO_2_ systems for CH_4_ detection has not yet been reported and represents a key gap in the bimetallic catalyst literature. Separately, PdPt bimetallic decoration of SnO_2_ nanoparticles has demonstrated industrial potential for formaldehyde sensing at 180 °C [49], confirming that the PdPt alloy strategy is transferable across multiple analytes. Beyond alloy engineering, galvanic-replacement synthesis has produced highly dispersive Pd on ZnO with improved CH_4_ sensing performance [50], offering a transferable deposition route applicable to SnO_2_ NW functionalisation. SnO_2_ systems for CH_4_ detection have not yet been reported and represent a key gap in the bimetallic catalyst literature.

### 6.3. Single-Atom Catalysis (SAC)

Single-atom catalysis, dispersing sub-nanometre-size Pd atoms on SnO_2_ surfaces rather than nanoclusters, represents the ultimate expression of catalytic efficiency and, possibly, RT action. At ultra-low loadings (0.07–0.2 wt% Pd), Pd atoms coordinated to SnO_2_ surface oxygen vacancies have interesting electronic properties. All atoms are catalytically accessible; thus, maximum metal utilisation is achieved. The lack of Pd–Pd bonds also prevents metallic shorting, and the isolated coordination to specific SnO_2_ surface sites may impart selectivity advantages foreign to nanoparticle systems. ALD stands today as the go-to deposition technique for SACs, with cycle-by-cycle loading control allowing sub-monolayer precision. Yan et al. demonstrated room-temperature CO detection using Pd- and Au-decorated alumina, highlighting the advantage of ALD for synthesising catalytically active thin films with precise thickness control [51].

### 6.4. UV/Photo-Assisted and Flexible Substrate Sensing

The activation of Pd-SnO_2_ NW sensors, with UV photons, can provide an efficient way to work at room temperature, as photogenerated electron–hole pairs are generated, which significantly speeds up the activation of surface oxygen and the kinetics of analyte oxidation. Miniaturised UV LED sources (peak emission 365–385 nm and power < 10 mW), integrated coplanar with sensor elements, can provide continuous photocatalytic RT sensing without the use of heater power. Xia et al. [2] utilised the visible-light method (470 nm LED, photon energy ≈ 2.64 eV versus 365 nm UV photon energy ≈ 3.40 eV and required photon energy reduction of ≈22%). This further reduces the required photon energy and extends the technology platform to solar-powered autonomous monitoring nodes that could be deployed at a mine portal or ventilation shafts.

The Pd-SnO_2_ NW active layers can be deposited using the flexible substrate integration on polyimide, PDMS, or carbon fibre woven substrates. This allows for conformal sensor integration into smart mining PPE (personal protective equipment), such as helmets, gloves, and body-worn sensor vests. The electrospinning is substrate-independent and can effectively deposit these SnO_2_ nanofibres directly onto flexible substrates. The subsequent decoration of Pd either through ALD or photochemical routes completes the sensing element, which does not require high-temperature processing, damaging the polymer substrates.

### 6.5. Edge-AI and Autonomous Sensor Networks

The integration of ultra-low-power Pd–SnO_2_ sensor networks and ASICs for on-sensor inference marks the cutting edge of smart mining safety. Through edge-AI processing of the sensor array outputs, ultra-low-power neural network accelerators such as the ARM Cortex-M series microcontrollers with TensorFlow Lite may one day enable rapid gas identification of gas species and their concentration, as well as anomaly detection without the cloud (e.g., off the network). This would be beneficial in deep underground areas where wireless propagation is unreliable. LSTM-based CH_4_ forecasting has exhibited 4.23% MAPE in field-deployed implementation [38]. Embedding models like these within on-sensor microcontrollers would enable a fully autonomous predictive safety monitoring system, requiring no human intervention between surface inspections. Current challenges, mitigation strategies, proposed directions, and expected outcomes for Pd–SnO_2_ NW methane sensor development are summarised in Table 5.

## 7. Conclusions

This mini-review synthesises about 60 peer-reviewed primary studies (2020–2026), drawn from systematic searches of Scopus, Web of Science and PubMed, with the aim to provide a critical account of the present and immediate future of Pd-decorated SnO_2_ nanowire systems for room-temperature methane sensing in mining safety applications. The following are the main conclusions.

The modification of SnO_2_ NWs by Pd occurs through a combination of two mechanisms, which include chemical spillover and electronic sensitisation. The chemical spillover sets in motion the Pd-catalysed dissociation and subsequent migration of reactive species to a variety of surface sites on SnO_2_. Electronic sensitisation is the term that describes the formation of a Schottky barrier at the Pd/SnO_2_ interface. When combined synergistically at optimal Pd loadings of 1.0–2.5 mol%, significant enhancements of 3–100× are observed over bare SnO_2_ at equal analyte concentrations and temperatures.

How the synthesis method is critical to the sensor architecture and performance:

- VLS Growth produces the highest crystalline perfection for 1D charge transport;
- Hydrothermal routes yield scalable nanoporous structures of surface area 60 m^2^/g;
- ALD provides the sub-nm conformal deposition of Pd for maximum junction density.

Electrospinning produces nanofibres compatible with flexible substrates for large-area coverage. The high-performance sensors for mining should be synthesised by using VLS/electrospinning ALD Pd post-decoration using a multi-step process.

The performance metrics that have received current validation are for instance, response values of 17.6–21.3 at 300–3000 ppm CH_4_ for Pd–SnO_2_ nanoporous and mesoporous systems (at 340–400 °C; note these are non-NW specific or RT outcome), response times of 3–74 s (3–12 s for thermally activated systems; 74 s for photo-assisted RT systems), and sub-ppm detection limits in H_2_ analogue systems which more than fulfil MSHA regulatory demands (1.0–2.5% alert/alarm thresholds, <30 s response). There is an essential gap: the direct demonstration of pure Pd–SnO_2_ nanowire performance for CH_4_ at room temperature; the same response values have not yet been reported, and that is the main scientific challenge for the field.

The main issues that restrict field deployment include humidity tolerance—for example, a 20–50% response drop at high-RH, long-term stability—which has only been shown for 30–90 days against a 6–12-month requirement, and selectivity over CH_4_/CO/H_2_ mixtures—all of which are currently being targeted by bimetallic decoration, core–shell nanoarchitectures, and ML-augmented multi-sensor arrays.

The combination of RT Pd–SnO_2_ NW sensors with a versatile communication platform, smart helmets, and edge AI processors positions these materials as enabling technologies for the next generation of autonomous, predictive underground mine safety networks with a socio-economic impact on circa 7 million coal miners globally [52].

The main remaining research gap is a direct demonstration that Pd–SnO_2_ NWs achieve sub-100 ppm RT CH_4_ detection with > 30 days stability. Such demonstration is achievable via synthesis advances (ALD-deposited PdPt SAC on VLS SnO_2_ NWs), UV/photo-activation, and field-validated drift-compensation algorithms. Closing this gap would position Pd–SnO_2_ NW sensors as a credible, intrinsically safe alternative to heated MOS devices—one capable of battery-powered, continuous deployment across underground coal mines globally.

## Figures and Tables

**Table 1:** Comparative summary of key synthesis strategies for Pd-decorated SnO_2_ nanowires and related nanostructures, benchmarked across scalability, morphology control, and key performance indicators.

Synthesis Method	Pd Loading	Morphology	Surface Area	Key Advantage	Selected Reference
Hydrothermal	0.5–2.5 mol%	Nanoporous NPs	~60 m^2^/g	Scalable, low-energy, tuneable porosity	Yao et al., 2020 [1]
VLS-CVD	1–5 nm clusters	Single-crystal NWs (50–200 nm)	High (1D geometry)	Directional charge transport	Cai et al., 2020 [10]
Electrospinning	0.5–2 wt%	Polycrystalline nanofibres	~143 m^2^/g	Large area, flexible substrates	Bulemo et al., 2021 [12]
ALD/Sputtering	0.5 nm film	Conformal nanofilm on SiNWs	~High	Sub-nm uniformity, sinter-resistant	Fang et al., 2025 [14]
Photochemical	0.2–1 mol%	NPs on nanospheres/NWs	Moderate	Alloy phase control (PdSn)	Gschwend et al., 2021 [17]
Flame Spray Pyrolysis	0.2 mol% (optimal)	Embedded NPs in SnO_2_	High SA	Continuous, scalable production	Jabłczyńska et al., 2024 [16]

H_2_ data are shown where direct RT CH_4_ NW data are unavailable; the catalytic mechanisms are considered mechanistically transferable but not equivalent. Surface area values are representative; actual values depend on specific synthesis parameters and precursor concentrations. NPs = nanoparticles; NWs = nanowires; SA = surface area.

**Table 2:** Mechanistic comparison of chemical sensitisation (spillover), electronic sensitisation (Schottky barrier), and their synergistic combination in Pd-decorated SnO_2_ sensing systems.

Feature	Chemical Sensitisation (Spillover)	Electronic Sensitisation (Schottky)	Synergistic (Both)
Primary Actor	Pd catalytic surface	Pd–SnO_2_ heterojunction interface	Dual Pd sites + junction
Gas Activation	C–H bond dissociation via Pd → spilt H/CH_3_	Work function mismatch widens EDL	Both pathways active simultaneously
Operating Temp	Can enable RT (especially with rGO, UV)	Generally, 200–400 °C; lowers with engineering	RT possible with optimised loading
Pd Loading Optimum	Low (<0.5 mol%): max active surface sites	~1.0–2.5 mol%: max Schottky interface	Typically, 1.0–2.5 mol% Pd optimal
Response Amplitude	Moderate alone (~3–10×)	High alone (~10–20×)	Highest (>20×, up to 100× [1,3])
Key Evidence	In situ XPS, Raman [9]	DFT calculations [3]	Combined spectroscopy + DFT

EDL = electric double layer; XPS = X-ray photoelectron spectroscopy; DFT = density functional theory; RT = room temperature (25 °C); rGO = reduced graphene oxide; UV = ultraviolet. Response amplitude multiples (×) are relative to bare SnO_2_ under identical measurement conditions.

**Table 4:** Assessment of Pd–SnO_2_ NW sensor technology against key mining safety application requirements, benchmarked against regulatory standards and current laboratory performance.

Parameter	Regulatory/Industrial Requirement	Pd–SnO_2_ NW Current Status	Gap/Comment
Alert Threshold	1.0% CH_4_ (MSHA early alert)	Achieved in lab (low ppm–%LEL)	✓ Demonstrated
Evacuation Limit	2.0% CH_4_ (30 CFR §75.323: withdrawal at 1.5%; full evacuation at 2.0%)	Full-range response confirmed	✓ Satisfied
LEL (Lower Explosive Limit)	5% CH_4_ in air	Sensors respond across range	✓ Covered
Power Consumption	<1 W (intrinsic safety)	RT sensors: 0 W heater power	✓ RT designs meet this
Response Time	<30 s (real-time alert)	3–74 s (3–12 s for thermally activated; 74 s for photo-assisted RT systems)	⚠ Thermally activated: ✓ (3–12 s); Photo-assisted RT: ✗ (74 s > 30 s limit)
Humidity Tolerance	RH 0–90% stable signal	Active area of improvement	✗ Needs hydrophobic coating
Long-term Stability	>6 months, no recalibration	30–60 days demonstrated (H_2_ analogues)	△ Requires extended validation
Selectivity (vs. CO, H_2_)	High specificity required	>80% reported with bimetals	△ Ongoing; bimetal helps

Notes: ✓ = Requirement met; ✗ = Requirement not met; ⚠ = Partially met (condition-dependent); △ = Under development/requires further validation. Abbreviations: MSHA, Mine Safety and Health Administration; LEL, Lower Explosive Limit; CFR, Code of Federal Regulations; RT, room temperature; NW, nanowire; RH, relative humidity; ppm, parts per million; %LEL, percent of lower explosive limit.

**Table 5:** Summary of current challenges in Pd-decorated SnO_2_ NW methane sensors for mining applications, current mitigation strategies, future research directions, and expected outcomes.

Challenge	Current Mitigation Strategy	Proposed Future Direction	Expected Outcome
Humidity interference (20–50% response drop)	Core–shell CeO_2_/SnO_2_ hydrophobic coating	Heterostructure hydrophobic shells	Stable operation at RH > 85%
Limited RT CH_4_ activity	rGO/CNT hybrids; UV photo-activation	UV-LED integrated micro-sensors	True RT, sub-ppm detection
Pd agglomeration & cost	ALD ultra-thin films; single-atom Pd	Single-atom catalysis (0.07 wt%)	Max atom efficiency, ppb LOD
Baseline drift (<10%/30 days)	Optimised calcination + encapsulation	Drift-correction AI algorithms	Self-calibrating sensor nodes
Selectivity vs. CO/H_2_	PdPt or PdAu bimetal decoration	Multi-gas sensor arrays + ML	91.6% gas classification accuracy [38]
Scalability for industrial mining	FSP and hydrothermal batch processes	Roll-to-roll ALD on flexible substrates	Mass-market wearable sensors

## Data Availability

No new data were created or analysed in this study. Data sharing is not applicable to this article.
